# Epilepsy and the risk of adverse cardiovascular events: A nationwide cohort study

**DOI:** 10.1111/ene.16116

**Published:** 2024-01-02

**Authors:** Josephine Mayer, Ameenathul M. Fawzy, Arnaud Bisson, Marco Pasi, Alexandre Bodin, Pascal Vigny, Julien Herbert, Anthony G. Marson, Gregory Y. H. Lip, Laurent Fauchier

**Affiliations:** ^1^ Liverpool Centre for Cardiovascular Science, University of Liverpool, Liverpool John Moores University, and Liverpool Heart and Chest Hospital Liverpool UK; ^2^ Walton Centre NHS Foundation Trust, Department of Pharmacology and Therapeutics Institute of Systems, Molecular, and Integrative Biology, University of Liverpool Liverpool UK; ^3^ Department of Cardiology Tours Regional University Hospital, Hospital Trousseau Tours France; ^4^ Department of Neurology, Hospital Bretonneau Tours France; ^5^ Clinical Data Centre, Public Health and Prevention Unit Tours University Hospital Tours France; ^6^ Danish Centre for Health Services Research, Department of Clinical Medicine Aalborg University Aalborg Denmark

**Keywords:** cardiovascular diseases, comorbidity, epilepsy, incidence, stroke

## Abstract

**Background and purpose:**

Epilepsy is associated with higher morbidity and mortality compared to people without epilepsy. We performed a retrospective cross‐sectional and longitudinal cohort study to evaluate cardiovascular comorbidity and incident vascular events in people with epilepsy (PWE).

**Methods:**

Data were extracted from the French Hospital National Database. PWE (*n* = 682,349) who were hospitalized between January 2014 and December 2022 were matched on age, sex, and year of hospitalization with 682,349 patients without epilepsy. Follow‐up was conducted from the date of first hospitalization with epilepsy until the date of each outcome or date of last news in the absence of the outcome. Primary outcome was the incidence of all‐cause death, cardiovascular death, myocardial infarction, hospitalization for heart failure, ischaemic stroke (IS), new onset atrial fibrillation, sustained ventricular tachycardia or fibrillation (VT/VF), and cardiac arrest.

**Results:**

A diagnosis of epilepsy was associated with higher numbers of cardiovascular risk factors and adverse cardiovascular events compared to controls. People with epilepsy had a higher incidence of all‐cause death (incidence rate ratio [IRR] = 2.69, 95% confidence interval [CI] = 2.67–2.72), cardiovascular death (IRR = 2.16, 95% CI = 2.11–2.20), heart failure (IRR = 1.26, 95% CI = 1.25–1.28), IS (IRR = 2.08, 95% CI = 2.04–2.13), VT/VF (IRR = 1.10, 95% CI = 1.04–1.16), and cardiac arrest (IRR = 2.12, 95% CI = 2.04–2.20). When accounting for all‐cause death as a competing risk, subdistribution hazard ratios for ischaemic stroke of 1.59 (95% CI = 1.55–1.63) and for cardiac arrest of 1.73 (95% CI = 1.58–1.89) demonstrated higher risk in PWE.

**Conclusions:**

The prevalence and incident rates of cardiovascular outcomes were significantly higher in PWE. Targeting cardiovascular health could help reduce excess morbidity and mortality in PWE.

## INTRODUCTION

Epilepsy is a common neurological condition, with lifetime prevalence reported to be 7.60/1000 persons [1]. Mortality in patients with epilepsy (PWE) has remained unchanged since the 1950s and is significantly higher compared to the general population [2, 3]. Only a small proportion of deaths in people with epilepsy are believed to be directly attributable to seizures, with the majority being secondary to epilepsy‐related causes such as structural brain abnormalities, stroke, neoplasms, and sudden unexpected death in epilepsy (SUDEP) [2, 3, 4]. Epilepsy‐unrelated causes such as cardiovascular disease are contributory, and studies have indicated a higher risk of cardiovascular events such as stroke, myocardial infarction (MI), and sudden cardiac death in PWE. Cardiovascular‐related deaths may account for approximately one quarter of deaths in PWE [2, 3, 5].

The relationship between cardiovascular disease and epilepsy is complex. The concept of the "epileptic heart" was proposed by Verrier et al. [4] to explicate the changes that occur in the myocardium and coronary vasculature as a result of the recurrent catecholaminergic surges and hypoxemia that occur with seizure activity in patients with chronic epilepsy. Over time, these cardiotoxic insults are believed to cause mechanical and electrical dysfunction, consequently increasing the risk of adverse cardiovascular outcomes such as arrhythmias and sudden cardiac death. It is hypothesized that there may be potential overlap between the latter and SUDEP [4]. Epilepsy is also a symptom of cardiovascular disease. Stroke is a common cause of adult onset epilepsy [6], and seizures in later life may be a presentation of subclinical cerebrovascular disease [7]. Other factors that influence cardiovascular health in PWE include lifestyle behaviors such as higher rates of smoking and socioeconomic deprivation, which has been associated with higher mortality in PWE [5, 8]. In addition, some antiseizure medications may be linked to accelerated atherosclerosis [9, 10, 11], but whether this translates to adverse cardiovascular outcomes in PWE is less clear [12].

At present, there are a limited number of studies specifically exploring cardiovascular outcomes such as mortality, arrhythmias, and cardiac arrest in PWE, despite the clinical implications. Thus, to gain a better understanding of the epidemiology of cardiovascular disease and outcomes in these patients, we performed a retrospective cross‐sectional and longitudinal cohort study to evaluate cardiovascular comorbidity and incident vascular events in hospitalized patients with epilepsy.

## METHODS

This study was carried out using the national hospitalization database Programme de Médicalisation des Systèmes d'Information (PMSI), which includes data on hospital care for the entire French population from January 2014 to December 2022. This was inspired by the US Medicare system and was implemented in 2004 so that all medical activity is recorded in a database, computed, and rendered anonymous. It includes more than 98% of the French population (67 million people) from birth (or immigration) to death (or emigration). This process allows the determination of each hospital's budget, in 1546 French health care facilities for both public and private hospitals.

Each hospitalization is encoded in a standardized dataset, which includes information about the patient (age and sex), hospital, stay (date of admission, date of discharge, and modes of discharge), pathologies, and procedures. Routinely collected medical information includes the principal diagnosis and secondary diagnoses. In the PMSI system, identified diagnoses are coded according to the International Classification of Diseases, Tenth Revision (ICD‐10). All medical procedures are recorded according to the national nomenclature, Classification Commune des Actes Medicaux. The PMSI contains individual anonymized information on each hospitalization that is linked to create a longitudinal record of hospital stays and diagnoses for each patient. The reliability of PMSI data has already been assessed [13, 14], and this database has previously been used to study patients with cardiovascular conditions [15, 16, 17]. There were no missing data, as the information was based on codes [15, 18].

The study was conducted retrospectively and, as patients were not involved in its conduct, there was no impact on their care. Ethical approval was not required, as all data were anonymized. The French Data Protection Authority granted access to the PMSI data. Procedures for data collection and management were approved by the Commission Nationale de l'Informatique et des Libertés, the independent national ethical committee protecting human rights in France, which ensures that all information is kept confidential and anonymous, in compliance with the Declaration of Helsinki (authorization number 1897139).

Patients with epilepsy aged >18 years were identified from hospital records if they had been admitted between 1 January 2014 and 31 December 2022 for any cause, and had been coded with a diagnosis of epilepsy (ICD‐10 G40 and G41). Patient information (demographics, comorbidities, medical history, and events during hospitalization or follow‐up) was obtained using data collected in the hospital records. For each hospital stay, combined diagnoses at discharge were obtained. Each variable was identified using ICD‐10 codes (see Table S1). For each patient with a history of epilepsy, a hospitalized patient matched on age, sex, and year of inclusion with no epilepsy was selected. Matching was performed using propensity scores, which were calculated using logistic regression with epilepsy as the dependent variable. The propensity score included age and sex. For each patient with epilepsy, a propensity score‐matched patient with no epilepsy was selected (1:1) using the one‐to‐one nearest neighbour method (with a caliper of 0.01 of the SD of the propensity score on the logit scale) and no replacement.

The occurrence of cardiovascular outcomes was reviewed within this period (1 January 2014 to 31 December 2022) and compared with hospitalized patients with no epilepsy matched for age, sex, and year of inclusion. Primary outcome was the incidence and risk of all‐cause death, cardiovascular death, and major cardiovascular events. We considered major cardiovascular events to be new MI, excluding patients with prior MI; hospitalization for heart failure; ischaemic stroke; new atrial fibrillation (AF), excluding patients with prior AF diagnosis; and sustained ventricular tachycardia or fibrillation (VT/VF). The endpoints were evaluated with follow‐up starting from the date of hospitalization from any cause with a new or existing diagnosis of epilepsy (or date of first hospitalization, matched with the year of inclusion, for patients with no epilepsy) until the date of each specific outcome or date of last news in the absence of the outcome. Information on outcomes during follow‐up was obtained by analyzing the PMSI codes for each patient. Outcomes were identified using their respective ICD‐10 codes (see Table S1). The mode of death (cardiovascular or noncardiovascular) was identified based on the main diagnosis during hospitalization resulting in death. To check for the persistence of associations after removal of cerebrovascular disease, a subanalysis was conducted excluding patients with prior ischaemic stroke and intracranial hemorrhage from the analysis.

### Statistical analysis

Qualitative variables are described as frequency and percentage and quantitative variables as mean and SD. Comparisons were made using *χ* ^2^ tests for categorical variables and the Student *t*‐test for continuous variables. The analysis for clinical outcomes during the whole follow‐up in the groups of interests was performed using the Mantel–Haenszel method to estimate standardized incidence rates and incidence rate ratios (IRRs) with 95% confidence intervals (CIs). A competing risk analysis was performed using the Fine and Gray model with subdistribution hazard ratios (SHRs) used to denote the risk of a particular outcome. The competing risk included noncardiovascular death for cardiovascular death, and all‐cause death for nonlethal clinical outcomes. There was no competing risk analysis for all‐cause death. All comparisons with *p* < 0.05 were considered statistically significant. All analyses were performed using Enterprise Guide 7.1, (SAS Institute, Cary, NC, USA).

## RESULTS

**FIGURE 1:**
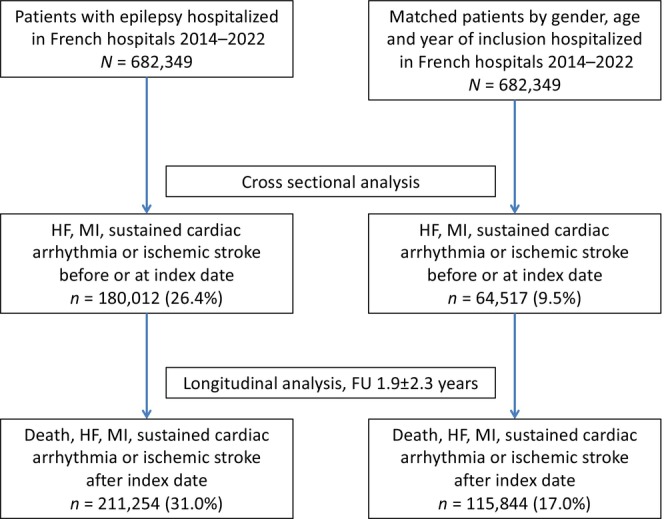
Flow chart of the matched study population. FU, follow‐up; HF, heart failure; MI, myocardial infarction.

A total of 682,349 hospitalized epilepsy patients were identified. They were matched on a 1:1 basis with 682,349 patients without epilepsy, according to age, gender, and year of first hospitalization within the time period. The study patient flow is shown in Figure 1.

### Baseline characteristics

Baseline characteristics and prevalent disease are demonstrated in Table 1. The mean age of the epilepsy and matched control group was 61.4 ± 20.5 years, with a range of 18–114 years. There was a slight male predominance of 52.8% (*n* = 360,485).

**TABLE 1:** Baseline medical diagnoses observed in patients with epilepsy compared to matched controls with no epilepsy.

Characteristic	No epilepsy, *n* = 682,349	Epilepsy, *n* = 682,349	*p*	Total, *n* = 1,364,698
Age, years, mean ± SD	61.4 ± 20.5	61.4 ± 20.5	1	61.4 ± 20.5
Age, years, median (IQR)	64.0 (47.0–78.0)	64.0 (47.0–78.0)		
Sex, male, *n* (%)	360,485 (52.8)	360,485 (52.8)	1	720,970 (52.8)
Cardiovascular risk factors and lifestyle behaviors, *n* (%)				
Hypertension	105,082 (15.4)	257,382 (37.7)	<0.0001	362,464 (26.6)
Diabetes mellitus	43,943 (6.4)	103,649 (15.2)	<0.0001	147,592 (10.8)
Chronic kidney disease	8734 (1.3)	37,052 (5.4)	<0.0001	45,786 (3.4)
Smoker	18,492 (2.7)	75,058 (11.0)	<0.0001	93,550 (6.9)
Dyslipidaemia	29,682 (4.4)	90,207 (13.2)	<0.0001	119,889 (8.8)
Obesity	29,546 (4.3)	72,465 (10.6)	<0.0001	102,011 (7.5)
Alcohol‐related diagnoses	14,398 (2.1)	95,529 (14.0)	<0.0001	109,926 (8.1)
Poor nutrition	23,405 (3.4)	120,708 (17.7)	<0.0001	144,112 (10.6)
Cardiovascular outcomes, *n* (%)				
Heart failure	29,750 (4.4)	86,522 (12.7)	<0.0001	116,272 (8.5)
Previous MI	8188 (1.2)	14,056 (2.1)	<0.0001	22,245 (1.6)
Atrial fibrillation	33,640 (4.9)	91,435 (13.4)	<0.0001	125,075 (9.2)
Previous VF/sustained VT	1474 (0.2)	5356 (0.8)	<0.0001	6830 (0.5)
Previous cardiac arrest	1760 (0.3)	10,372 (1.5)	<0.0001	12,132 (0.9)
Ischaemic stroke	8052 (1.2)	60,524 (8.9)	<0.0001	68,576 (5.0)
Other cardiovascular‐related disorders, *n* (%)				
History of pulmonary oedema	1494 (0.2)	7574 (1.1)	<0.0001	9068 (0.7)
Valve disease	9962 (1.5)	29,409 (4.3)	<0.0001	39,372 (2.9)
Aortic stenosis	4852 (0.7)	13,101 (1.9)	<0.0001	17,953 (1.3)
Aortic regurgitation	1467 (0.2)	5868 (0.9)	<0.0001	7335 (0.5)
Mitral regurgitation	3480 (0.5)	11,941 (1.8)	<0.0001	15,421 (1.1)
Dilated cardiomyopathy	4510 (0.7)	16,445 (2.4)	<0.0001	20,955 (1.5)
Coronary artery disease	33,503 (4.9)	70,828 (10.4)	<0.0001	104,331 (7.6)
Previous PCI	4183 (0.6)	12,896 (1.9)	<0.0001	17,079 (1.3)
Previous CABG	21 (0.0)	1788 (0.3)	<0.0001	1809 (0.1)
Previous pacemaker or ICD	6496 (1.0)	21,767 (3.2)	<0.0001	28,263 (2.1)
Vascular disease	23,200 (3.4)	68,098 (10.0)	<0.0001	91,298 (6.7)
Intracranial bleeding	4272 (0.6)	60,320 (8.8)	<0.0001	64,591 (4.7)
Noncardiovascular pathology, *n* (%				
Lung disease	21,835 (3.2)	109,790 (16.1)	<0.0001	131,625 (9.6)
Sleep apnea syndrome	9826 (1.4)	31,388 (4.6)	<0.0001	41,214 (3.0)
COPD	10,645 (1.6)	41,487 (6.1)	<0.0001	52,131 (3.8)
Liver disease	6557 (1.0)	37,461 (5.5)	<0.0001	44,018 (3.2)
Thyroid diseases	16,240 (2.4)	53,019 (7.8)	<0.0001	69,258 (5.1)
Inflammatory disease	11,805 (1.7)	34,390 (5.0)	<0.0001	46,195 (3.4)
Anaemia	20,470 (3.0)	92,731 (13.6)	<0.0001	113,202 (8.3)
Previous cancer	43,807 (6.4)	112,110 (16.4)	<0.0001	155,917 (11.4)
Cognitive impairment	19,038 (2.8)	97,098 (14.2)	<0.0001	116,136 (8.5)

*Note*: Values are *n* (%) or mean ± SD.

Abbreviations: CABG, coronary artery bypass graft; COPD, chronic obstructive pulmonary disease; ICD, implantable cardioverter defibrillator; IQR, interquartile range; MI, myocardial infarction; PCI, percutaneous coronary intervention; VF, ventricular fibrillation; VT, ventricular tachycardia.

Cardiovascular risk factors and comorbidities such as hypertension, dyslipidaemia, diabetes, coronary artery disease, valvulopathy, heart failure, and ischaemic stroke were more prevalent in the epilepsy group. They were also more likely to have arrhythmias such as AF, VT/VF, and previous cardiac arrests, although the latter only comprised a small proportion of the cohort. Noncardiovascular comorbidities such as lung pathologies, malignancy, and thyroid and inflammatory diseases were also more common in PWE. In addition, PWE had a higher prevalence of poorer lifestyle‐related factors including poor nutrition, obesity, smoking, and alcohol‐related disorders.

### Cross‐sectional analysis

In the cross‐sectional analysis, which looked at events at or before the index date, 180,012 events of heart failure, MI, sustained cardiac arrhythmia, and ischaemic stroke had occurred in the epilepsy group, and 64,517 events had occurred in the control group at the time of inclusion in the study.

### Longitudinal analysis

**FIGURE 2:**
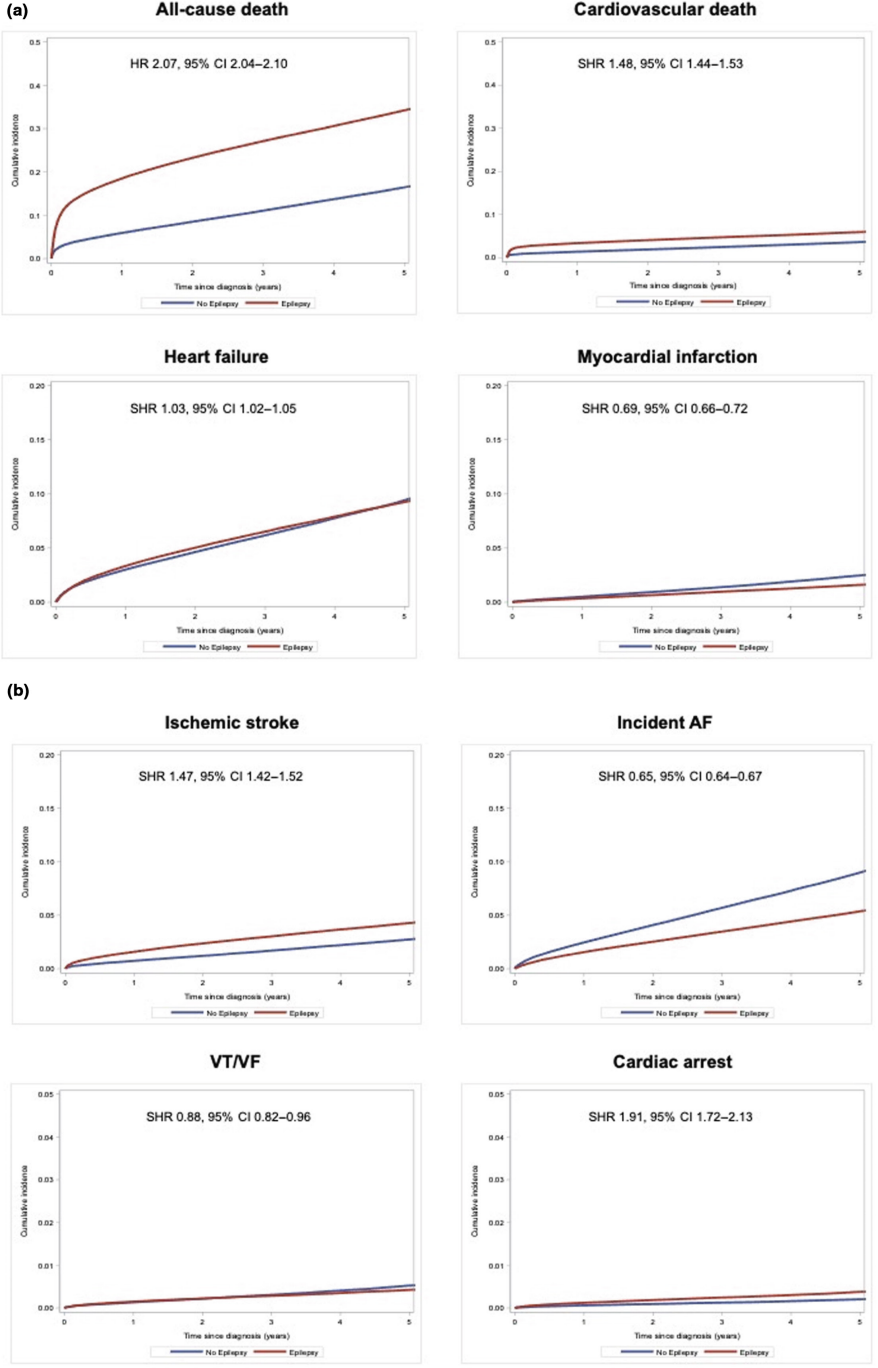
(a) Cumulative incidence function curves for clinical outcomes with censoring for competing events in patients with and without history of epilepsy: all‐cause death (left upper panel), cardiovascular death (right upper panel), hospitalization for heart failure (left lower panel), and myocardial infarction (right lower panel). (b) Cumulative incidence function curves for clinical outcomes with censoring for competing events in patients with and without history of epilepsy: ischaemic stroke (left upper panel), atrial fibrillation (AF; right upper panel), incident ventricular tachycardia or fibrillation (VT/VF; left lower panel), and incident cardiac arrest (right lower panel). CI, confidence interval; HR, hazard ratio; SHR, subdistribution hazard ratio.

Over a mean follow‐up duration of 1.9 ± 2.3 years, 327,098 incident events were observed in both groups; 211,254 events in the epilepsy group and 115,844 events in the control group. The incidence rates for each of these outcomes are presented in Table 2. Figure 2 demonstrates the cumulative incidence function curves for the outcomes.

**TABLE 2:** Incident outcomes in the age‐ and sex‐matched population according to history of epilepsy or no epilepsy.

Outcome	No epilepsy, *n* = 682,349			Epilepsy, *n* = 682,349						
							Incidence rate ratio (95% CI)	*p*	Subdistribution hazard ratio (95% CI)	*p*
	Person‐time (patient‐ years)	Events, *n*	Incidence, %/year (95% CI)	Person‐time (patient‐ years)	Events, *n*	Incidence, %/year (95% CI)				
All‐cause death	1,347,819	56,638	4.20 (4.17–4.24)	1,302,399	147,335	11.31 (11.26–11.37)	2.70 (2.67–2.72)	<0.0001	2.50 (2.47–2.52)	<0.0001
Cardiovascular death	1,347,819	12,605	0.94 (0.92–0.95)	1,302,399	26,277	2.02 (1.99–2.04)	2.16 (2.11–2.20)	<0.0001	1.79 (1.75–1.83)	<0.0001
Heart failure	1,288,611	48,474	3.76 (3.73–3.80)	1,232,982	58,506	4.75 (4.71–4.78)	1.26 (1.25–1.28)	<0.0001	0.97 (0.95–0.98)	<0.0001
Incident MI	1,334,162	10,317	0.77 (0.76–0.79)	1,291,671	8505	0.66 (0.65–0.67)	0.85 (0.83–0.88)	<0.0001	0.63 (0.61–0.65)	<0.0001
Ischaemic stroke	1,334,393	12,198	0.91 (0.90–0.93)	1,270,941	24,209	1.91 (1.88–1.93)	2.08 (2.04–2.13)	<0.0001	1.59 (1.55–1.63)	<0.0001
Incident AF	1,291,626	40,096	3.10 (3.07–3.14)	1,262,210	29,309	2.32 (2.30–2.35)	0.75 (0.74–0.76)	<0.0001	0.58 (0.57–0.59)	<0.0001
VT/VF	1,345,082	2285	0.17 (0.16–0.18)	1,299,423	2422	0.19 (0.18–0.19)	1.10 (1.04–1.16)	0.002	0.83 (0.78–0.89)	<0.0001
Cardiac arrest	1,347,182	4209	0.31 (0.30–0.32)	1,300,037	8591	0.66 (0.65–0.68)	2.12 (2.04–2.20)	<0.0001	1.73 (1.58–1.89)	<0.0001

Abbreviations: AF, atrial fibrillation; CI, confidence interval; MI, myocardial infarction; VT/VF, ventricular tachycardia or fibrillation.

### All‐cause death

A total of 147,335 events were observed in the epilepsy group, and 56,638 events were observed in the control group, with corresponding incidence rates of 11.31%/year and 4.20%/year, IRR 2.63 (95% CI = 2.67–2.72). SHR for all‐cause death was 2.50 (95% CI = 2.47–2.52), demonstrating a nearly threefold higher risk in the epilepsy group.

### Cardiovascular death

A total of 26,277 and 12,605 cardiovascular deaths occurred in the epilepsy and control groups, respectively. The incidence (IRR = 2.16, 95% CI = 2.11–2.20) and risk (SHR = 1.79, 95% CI = 1.75–1.83) of cardiovascular death were significantly higher in the epilepsy group.

### Heart failure

Incident heart failure events in the epilepsy and control cohorts were 58,506 and 48,474, respectively. The incidence of heart failure was significantly higher in the epilepsy group (IRR = 1.26, 95% CI = 1.25–1.28). In the competing risk analysis, the risk of incident heart failure was significantly lower in the epilepsy group (SHR = 0.97, 95% CI = 0.93–0.98).

### Myocardial infarction

Fewer incident MIs were observed in the epilepsy group compared to the control group (7043 vs. 8223). The incidence (IRR = 0.89, 95% CI = 0.86–0.91) and risk (SHR = 0.63, 95% CI = 0.61–0.65) of MI were significantly lower in the control group compared to the epilepsy group.

### Ischaemic stroke

A total of 24,209 ischaemic stroke events were observed in the epilepsy group, and 12,198 events were observed in the control group. The incidence of ischaemic stroke was twofold higher in the epilepsy group (IRR = 2.08, 95% CI = 2.04–2.13), and subdistribution hazard demonstrated significantly increased risk in the epilepsy group (SHR = 1.59, 95% CI = 1.55–1.63).

### Atrial fibrillation

A total of 29,309 patients had AF in the epilepsy group, and 40,096 in the control group had incident AF. The incidence (IRR 0.75, 95% CI = 0.74–0.76) and risk (SHR = 0.58, 95% CI = 0.57–0.59) of incident AF were significantly lower in the epilepsy cohort compared to the nonepilepsy cohort.

### Ventricular tachycardia or fibrillation

VT/VF events in the epilepsy and control groups were 2422 and 2285, respectively, with an IRR of 1.10 (95% CI = 1.04–1.16) indicating a higher rate of ventricular arrhythmias in the epilepsy group. The SHR of 0.834 (95% CI = 0.780–0.893) was shown to be significantly lower in this cohort after accounting for the competing event of all‐cause death.

### Cardiac arrests

A total of 8591 cardiac arrests were observed in the epilepsy group, and 4209 were observed in the control group. Incidence of cardiac arrests was twofold higher in the epilepsy group (IRR = 2.12, 95% CI = 2.04–2.20), and risk was also significantly elevated (SHR = 1.73, 95% CI = 1.58–1.89) compared to controls.

### Exploratory analysis

Ischaemic stroke and intracranial hemorrhage are the most common vascular disease‐related causes of epilepsy. We performed a subgroup analysis excluding patients with a prior history of ischaemic stroke and intracranial hemorrhage (Table 3). The baseline comorbidities of the groups are outlined in Table S2. Results were similar to our primary analysis, with elevated incidence of cardiovascular death (IRR = 1.72, 95% CI = 1.67–1.77), heart failure (IRR = 1.26, 95% CI = 1.25–1.28), ischaemic stroke (IRR = 1.76, 95% CI = 1.71–1.80), VT/VF (IRR = 1.08, 95% CI = 1.01–1.15), and cardiac arrest (IRR = 2.18, 95% CI = 2.10–2.28) in PWE compared to controls, whereas the incident rate and risk of MI and AF remained higher in the control group.

**TABLE 3:** Incident outcomes in the age‐ and sex‐matched population according to history of epilepsy with no history of stroke or intracranial bleeding, or no epilepsy.

Outcome	No epilepsy, *n* = 571,532			Epilepsy, *n* = 571,532						
							Incidence rate ratio (95% CI)	*p*	Subdistribution hazard ratio (95% CI)	*p*
	Person‐time (patient‐ years)	Events, *n*	Incidence, %/year (95% CI)	Person‐time (patient‐ years)	Events, *n*	Incidence, %/year (95% CI)				
All‐cause death	1,128,364	42,446	3.76 (3.73–3.80)	1,143,369	114,379	10.00 (9.95–10.06)	2.66 (2.63–2.69)	<0.0001	2.51 (2.49–2.54)	<0.0001
Cardiovascular death	1,128,364	8617	0.76 (0.75–0.78)	1,143,369	15,030	1.32 (1.29–1.34)	1.72 (1.67–1.77)	<0.0001	1.47 (1.43–1.51)	<0.0001
Heart failure	1,082,357	37,262	3.44 (3.41–3.48)	1,085,943	47,185	4.35 (4.31–4.38)	1.26 (1.25–1.28)	<0.0001	0.98 (0.97–1.00)	0.04
Incident MI	1,117,661	8133	0.73 (0.71–0.74)	1,134,256	6975	0.62 (0.60–0.63)	0.85 (0.82–0.87)	<0.0001	0.64 (0.61–0.66)	<0.0001
Ischaemic stroke	1,119,326	8419	0.75 (0.74–0.77)	1,124,269	14,839	1.32 (1.30–1.34)	1.76 (1.71–1.80)	<0.0001	1.37 (1.33–1.42)	<0.0001
Incident AF	1,084,524	31,069	2.87 (2.83–2.90)	1,108,888	24,599	2.22 (2.19–2.25)	0.77 (0.76–0.79)	<0.0001	0.60 (0.59–0.62)	<0.0001
VT/VF	1,126,190	1809	0.16 (0.15–0.17)	1,140,863	1981	0.17 (0.17–0.18)	1.08 (1.01–1.15)	0.02	0.83 (0.77–0.90)	<0.0001
Cardiac arrest	1,127,880	3302	0.29 (0.28–0.30)	1,141,261	7299	0.64 (0.63–0.65)	2.18 (2.10–2.28)	<0.0001	1.76 (1.59–1.91)	<0.0001

Abbreviations: AF, atrial fibrillation; CI, confidence interval; MI, myocardial infarction; VT/VF, ventricular tachycardia or fibrillation.

## DISCUSSION

In this large population‐based observational study of associated comorbidity and incident vascular outcomes in PWE, we observed that in hospitalized patients, a diagnosis of epilepsy was associated with (i) an increased prevalence of cardiovascular risk factors such as hypertension, diabetes, and dyslipidaemia; (ii) a higher prevalence of cardiovascular comorbidities; (iii) a higher prevalence of poorer lifestyle behaviors, including smoking, obesity, alcohol use, and poor diet; and (iv) a significantly higher incidence of death from all causes including cardiovascular death, heart failure, ischaemic stroke, VT/VF, and cardiac arrest.

To our knowledge, this is the largest study of comorbidity and incident cardiovascular outcomes in PWE. This work does not imply causation, but generates a number of hypotheses that warrant attention, including the possibility of shared mechanisms between cardiac and brain health. Our findings reflect results from other observational studies that have identified a higher prevalence of cardiac comorbidities, vascular risk factors, poorer lifestyle behaviors, and increased rates of cardiac mortality, morbidity, and stroke in PWE [12, 19, 20, 21, 22, 23, 24, 25].

The cross‐sectional analysis of the epilepsy group differed significantly for cardiovascular risk factors, outcomes, and poorer lifestyle behaviors. These factors are likely contributory to the high incident rates of cardiovascular outcomes. The risk of cardiovascular disease, however, has remained elevated in other observational cohorts even after adjustment for patients with prior stroke and vascular risk factors [12, 21, 25, 26], providing evidence towards an additional contribution of epilepsy‐related factors to adverse cardiovascular outcomes. In this study, when we removed patients with prior stroke diagnoses to remove bias towards an association with cardiovascular disease, PWE still had elevated incident rates of heart failure, ischaemic stroke, VT/VF, and cardiovascular death.

Poorer lifestyle behaviors and socioeconomic factors [8, 27] are likely to be important contributors to cardiovascular health in PWE. In the cross‐sectional analysis in this study, PWE had significantly higher levels of smoking, poor diet, obesity, and alcohol‐related disorders. Terman et al. identified elevated cardiovascular risk in PWE, which was attenuated after adjusting for lifestyle factors, suggesting targeting these may be an appropriate strategy to improve vascular health in this group of patients [28].

There are likely to be differing mechanisms underlying the association of adverse cardiovascular outcomes in those with epilepsy from a young age compared to those with late onset unprovoked epilepsy, where a shared vasculopathy may be contributory [7, 29], but data regarding mechanisms within different etiologies are scarce.

Contrasting with previous reports [21], in this French population we did not identify an increased incidence of MI in PWE. The incidence of MI has been reported as 24% higher in PWE compared to those with lower limb fracture in a South Carolina‐based study [22], and a correlation between number of hospitalizations with epilepsy, a proxy for seizure frequency, and risk of MI in PWE has been previously described [30]. In this study, where the mean participants' age was 61.4 ± 20.5 years, there was a higher prevalence of AF (13.4%) compared to the figures reported in some of the other studies [31]. When patients with prior AF diagnoses were removed from analysis, the incident rate was lower in the epilepsy group. It is well known that the incidence of AF increases with age, and it is possible that these figures may differ depending on the age of the study population [32]. Furthermore, given that cardiac arrhythmias can masquerade as epilepsy, an element of AF underreporting may also be present in PWE [33]. AF may also be more frequently diagnosed in the community [34]. Conversely, an association with a diagnosis of AF and the subsequent development of epilepsy (hazard ratio = 1.51, 95% CI = 1.35–1.69, *p* < 0.001) has also been previously reported [35].

In this study, there was an unexpected lower incidence of MI and AF in PWE. As patients with prior diagnoses were excluded from the incident analysis for MI and AF, the cohorts may not have reflected the cross‐sectional analysis. Furthermore, there are unknown medication and procedural factors (e.g., secondary prevention for stroke and heart failure management) that may modify patients' subsequent MI or AF risk. In addition, patients were not matched on comorbidities, which may lead to bias. As we do not know the details of cardiovascular disease deaths, the high risk of cardiovascular‐related deaths may be attributed to other aetiologies such as ischaemic stroke, cardiac arrest, or heart failure. There are also a number of other limitations in the study relating to the difficulty in obtaining an unbiased comparison of two clinically complex cohorts discussed below. Further studies examining this risk in those without a preexisting cardiovascular history may help clarify these relationships.

### Strengths and limitations

The strengths of the study include the size of the analysis and the coverage of the French hospitalized population. This study has a number of limitations due to the retrospective design and use of a large national dataset. There is a selection bias into the cohort, as both epilepsy and cardiovascular outcomes may lead to hospitalizations, which may result in biased associations between epilepsy and cardiovascular outcomes. There is a lack of detail regarding epilepsy diagnosis such as etiology and length of epilepsy diagnosis, and we cannot ascertain the finer details of the relationship of epilepsy with cardiovascular outcomes. A high death rate was seen in PWE in the cohort; this may reflect that the cohort only included hospitalized patients, which may result in a high number of those with symptomatic epilepsy. As diagnoses were based on clinical codes, they are subject to misclassification error, and therefore patients who may otherwise not have met diagnostic criteria for epilepsy may be included in the analysis; however, the ICD‐10 clinical codes for epilepsy have been demonstrated to have high accuracy in health care administrative datasets [36]. The consequences of antiseizure medication use are unaccounted for, and these are likely to contribute to poorer cardiovascular health in PWE via effects on cardiac electrophysiology and accelerated atherosclerotic disease [9, 12, 37]. Patients in the reference group were not censored if they were subsequently diagnosed with epilepsy, although these numbers are likely to be small. We were not able to adjust for shared‐risk factors or account for informative censorship. Furthermore, the population studied is a hospitalized population and does not consider PWE managed in the community, or illness or death that occurred in a nonhospital setting. We used the Fine and Gray model to produce subdistribution hazard ratios; this method has a number of limitations, and results should be interpreted with caution given that patients who died "unnaturally" remain in the at‐risk pool, and therefore a competing event may falsely protect individuals from the event of interest [38]. Given the limitations, we only conclude an association between epilepsy and adverse cardiovascular events in hospitalized patients that requires further investigation.

## CONCLUSIONS

The prevalence and incident rates of cardiovascular outcomes were significantly higher in hospitalized PWE. As cardiovascular disease contributes a significant proportion of morbidity and mortality in this population, improving cardiovascular health is an attractive target to reduce premature deaths in PWE.

## AUTHOR CONTRIBUTIONS

**Josephine Mayer:** Writing – original draft; writing – review and editing; methodology. **Ameenathul M. Fawzy:** Writing – original draft; writing – review and editing; methodology. **Arnaud Bisson:** Conceptualization; writing – review and editing; methodology. **Marco Pasi:** Conceptualization; methodology. **Alexandre Bodin:** Conceptualization; methodology. **Pascal Vigny:** Formal analysis; project administration; data curation; software. **Julien Herbert:** Software; formal analysis; project administration; data curation. **Anthony G. Marson:** Writing – review and editing; writing – original draft. **Gregory Y.H. Lip:** Conceptualization; writing – original draft; writing – review and editing. **Laurent Fauchier:** Conceptualization; investigation; methodology; formal analysis; writing – review and editing; project administration; resources; supervision; data curation; software.

## CONFLICT OF INTEREST STATEMENT

J.M. receives funding from the Association of British Neurologists, the Stroke Association, and Epilepsy Research UK as part of the Association of British Neurologists Clinical Research Fellowship scheme. A.Bi. has been a consultant and speaker for Medtronic, AstraZeneca, BMS/Pfizer, and Bayer. A.G.M. is a National Institute for Health Research (NIHR) Senior Investigator and also in part funded by NIHR ARC North West Coast. The views expressed in this article are those of the authors and not necessarily those of the NIHR or the Department of Health and Social Care. G.H.Y.L. has been a consultant and speaker for BMS/Pfizer, Medtronic, Boehringer Ingelheim, and Daiichi‐Sankyo. No fees are directly received personally. G.H.Y.L. is co‐principal investigator of the AFFIRMO project on multimorbidity in AF, which has received funding from the European Union's Horizon 2020 research and innovation program under grant agreement 899871. L.F. has been a consultant and speaker for AstraZeneca, Bayer, BMS/Pfizer, Boehringer Ingelheim, Medtronic, Novartis, Novo, XO, and Zoll. The remaining authors have no conflicts of interest.

## INSTITUTIONAL REVIEW BOARD STATEMENT

The study was conducted in accordance with the Declaration of Helsinki, and approved by an independent national ethics committee. Ethical approval was not required, as the study was retrospective and conducted using anonymized data without direct patient involvement.

## INFORMED CONSENT

Informed consent was not required, as the study was conducted retrospectively using anonymized data and without direct patient involvement.

## Supporting information


TABLE S1

## Data Availability

The data used in this study may be available from the corresponding author upon reasonable request.
