# Supplementary material for: Epilepsy and the risk of adverse cardiovascular events: A nationwide cohort study
Source: Eur J Neurol. 2024 Jan 2;31(3):e16116. doi: 10.1111/ene.16116 (PMC11235735; doi:10.1111/ene.16116)
Supplement: Supplementary file 1 — TABLE S1 [file ENE-31-e16116-s001.docx]

**Supplementary Table 1.** Codes in the 10th revision of the International Classification of Diseases (ICD-10) or in the classification commune des actes médicaux (CCAM, French classification for medical procedures) for diseases and comorbidities.

| **Diseases and comorbidities** | **Codes in ICD-10 or CCAM** |
| --- | --- |
| **Hypertension** | I10, I11, I12, I13, I14, I15 |
| **Diabetes mellitus** | E10, E11, E12, E13, E14 |
| **Heart failure** | I50, I11.0, I13.0, I13.2, J81, R570, I42, I43, I25.5 |
| **Pulmonary oedema** | R570, J81 |
| **Aortic stenosis** | I350, I352, I060, I062 |
| **Aortic regurgitation** | I35.1 |
| **Mitral regurgitation** | I34.0 |
| **Endocarditis** | I330 |
| **Dilated cardiomyopathy** | I42, I43 |
| **Coronary artery disease** | I20, I21, I22, I23, I24, I25,  Z955, DDAF006, DDAF001, DDAF003, DDAF004, DDAF007, DDAF008, DDAF009, DDAF010, Z951, DDMA003, DDMA004, DDMA005, DDMA006, DDMA007, DDMA008, DDMA009, DDMA011, DDMA012, DDMA013, DDMA015, DDMA016, DDMA017, DDMA018, DDMA019, DDMA020, DDMA021, DDMA022, DDMA023, DDMA024, DDMA025, DDMA026, DDMA027, DDMA028, DDMA029, DDMA030, DDMA031, DDMA032, DDMA033, DDMA034, DDMA035, DDMA036, DDMA037, DDMA038 |
| **Myocardial infarction** | I21, I22, I23 |
| **PCI** | Z955, DDAF006, DDAF001, DDAF003, DDAF004, DDAF007, DDAF008, DDAF009, DDAF010 |
| **CABG** | Z951, DDMA003, DDMA004, DDMA005, DDMA006, DDMA007, DDMA008, DDMA009, DDMA011, DDMA012, DDMA013, DDMA015, DDMA016, DDMA017, DDMA018, DDMA019, DDMA020, DDMA021, DDMA022, DDMA023, DDMA024, DDMA025, DDMA026, DDMA027, DDMA028, DDMA029, DDMA030, DDMA031, DDMA032, DDMA033, DDMA034, DDMA035, DDMA036, DDMA037, DDMA038 |
| **Atrial fibrillation** | I48 |
| **Left BBB** | I447 |
| **Right BBB** | I451 |
| **Ventricular tachycardia** | I47.2 |
| **Ventricular fibrillation** | I490 |
| **Cardiac arrest** | I46 |
| **Vascular disease** | I71, I790, I739, R02, Z958, Z959 |
| **Ischaemic stroke** | I63 |
| **Intracranial bleeding** | I60, I61, I62, S06.4, S06.5, S06.6 |
| **Smoker** | I472 |
| **Dyslipidaemia** | E78 |
| **Obesity** | E65, E66 |
| **Alcohol-related diagnoses** | E24.4, F10, G31.2, G62.1, G72.1, I42.6, K29.2, K70, K86.0, O35.4, P04.3, Q86.0, T51, Y90, Y91, Z50.2, Z71.4, Z72.1 |
| **Poor nutrition** | E41, E43, E44, E46, F508, K912, R636 |
| **Abnormal renal function** | N18.3, N18.4, N18.5, T86.1, Z49, Z99.2 |
| **Lung disease** | J40-J70, J96.1 |
| **Sleep apnoea syndrome** | G47.3 |
| **COPD** | J43, J44 |
| **Liver disease** | K70-K77 |
| **Thyroid diseases** | E0, E89.0 |
| **Inflammatory disease** | M05-M14, M45, M46, K50, K51, K52 |
| **Anaemia** | D50-D64 |
| **Previous cancer** | C00-C97 |
| **Cognitive impairment** | F00, F01, F02, F03, F106, F1073, F1173, F1273, F1373, F1473, F1573, F1673, F1773, F1873, F1973, G30, G310, G318, G938, I673 |

CABG = coronary artery bypass graft; COPD = chronic obstructive pulmonary disease; PCI = percutaneous coronary intervention.

**Supplementary Table 2**. Baseline medical diagnoses observed in patients with epilepsy (excluding prior history of ischemic stroke or intracranial hemorrhage) compared to matched controls with no epilepsy.

|  | **No Epilepsy** | **Epilepsy** | **p** |
| --- | --- | --- | --- |
|  | **(n=571532)** | **(n=571532)** |  |
| **Age (years), mean ± SD** | 59.5±20.7 | 59.5±20.7 | 1.00 |
| **Sex (male), n (%)** | 299311 (52.4) | 299311 (52.4) | 1.00 |
| ***Cardiovascular risk factors and lifestyle behaviors*** | | | |
| **Hypertension, n (%)** | 79672 (13.9) | 188263 (32.9) | <0.0001 |
| **Diabetes mellitus, n (%)** | 34235 (6.0) | 77557 (13.6) | <0.0001 |
| **Chronic kidney disease, n (%)** | 6630 (1.2) | 27262 (4.8) | <0.0001 |
| **Smoker, n (%)** | 15489 (2.7) | 60297 (10.6) | <0.0001 |
| **Dyslipidemia, n (%)** | 22461 (3.9) | 62526 (10.9) | <0.0001 |
| **Obesity, n (%)** | 24747 (4.3) | 57553 (10.1) | <0.0001 |
| **Alcohol related diagnoses, n (%)** | 12002 (2.1) | 79157 (13.9) | <0.0001 |
| **Poor nutrition, n (%)** | 17717 (3.1) | 91159 (16.0) | <0.0001 |
| ***Cardiovascular outcomes*** | | | |
| **Heart failure, n (%)** | 22861 (4.0) | 62526 (10.9) | <0.0001 |
| **Previous MI, n (%)** | 6573 (1.2) | 10116 (1.8) | <0.0001 |
| **Atrial fibrillation, n (%)** | 24519 (4.3) | 60697 (10.6) | <0.0001 |
| **Previous VF / sustained VT, n (%)** | 1149 (0.2) | 3984 (0.7) | <0.0001 |
| **Previous cardiac arrest, n (%)** | 1309 (0.2) | 8573 (1.5) | <0.0001 |
| **Ischemic stroke, n (%)** | 0 (0.0) | 0 (0.0) | N/A |
| ***Other cardiovascular related disorders*** | | | |
| **History of pulmonary edema, n (%)** | 1120 (0.2) | 5607 (1.0) | <0.0001 |
| **Previous pacemaker or ICD, n (%)** | 4904 (0.9) | 15831 (2.8) | <0.0001 |
| **Valve disease, n (%)** | 7487 (1.3) | 20461 (3.6) | <0.0001 |
| **Aortic stenosis, n (%)** | 3669 (0.6) | 9202 (1.6) | <0.0001 |
| **Aortic regurgitation, n (%)** | 1080 (0.2) | 3961 (0.7) | <0.0001 |
| **Mitral regurgitation, n (%)** | 2640 (0.5) | 8173 (1.4) | <0.0001 |
| **Dilated cardiomyopathy, n (%)** | 3503 (0.6) | 11545 (2.0) | <0.0001 |
| **Coronary artery disease, n (%)** | 26290 (4.6) | 51324 (9.0) | <0.0001 |
| **Previous PCI, n (%)** | 3441 (0.6) | 9087 (1.6) | <0.0001 |
| **Previous CABG, n (%)** | 16 (0.0) | 1172 (0.2) | <0.0001 |
| **Vascular disease, n (%)** | 17603 (3.1) | 45665 (8.0) | <0.0001 |
| **Intracranial bleeding, n (%)** | 0 (0.0) | 0 (0.0) | N/A |
| ***Non cardiovascular pathology*** | | | |
| **Lung disease, n (%)** | 16860 (3.0) | 82701 (14.5) | <0.0001 |
| **Sleep apnea syndrome, n (%)** | 8116 (1.4) | 24747 (4.3) | <0.0001 |
| **COPD, n (%)** | 8344 (1.5) | 33606 (5.9) | <0.0001 |
| **Liver disease, n (%)** | 5418 (0.9) | 30634 (5.4) | <0.0001 |
| **Thyroid diseases, n (%)** | 12859 (2.3) | 41722 (7.3) | <0.0001 |
| **Inflammatory disease, n (%)** | 9830 (1.7) | 26233 (4.6) | <0.0001 |
| **Anemia, n (%)** | 16232 (2.8) | 71956 (12.6) | <0.0001 |
| **Previous cancer, n (%)** | 35492 (6.2) | 94417 (16.5) | <0.0001 |
| **Cognitive impairment, n (%)** | 13888 (2.4) | 74756 (13.1) | <0.0001 |

Values are n (%) or mean ± SD. CABG=coronary artery bypass graft; COPD = chronic obstructive pulmonary disease; ICD = implantable cardioverter defibrillator; MI = myocardial infarction; PCI=percutaneous coronary intervention; SD=standard deviation.
